# Electronic Medical Record System Use and Determinants in Ethiopia: Systematic Review and Meta-Analysis

**DOI:** 10.2196/40721

**Published:** 2023-01-11

**Authors:** Masresha Derese Tegegne, Sisay Maru Wubante, Mulugeta Hayelom Kalayou, Mequannent Sharew Melaku, Binyam Tilahun, Tesfahun Melese Yilma, Hiwote Simane Dessie

**Affiliations:** 1 Department of Health Informatics Institute of Public Health, College of Medicine and Health Sciences University of Gondar Gondar Ethiopia; 2 Department of Health Informatics School of Public Health, College of Medicine and Health Sciences Wollo University Dessie Ethiopia; 3 Department of Health Informatics School of Public Health, College of Medicine and Health Sciences Wachemo University Hosaena Ethiopia

**Keywords:** electronic medical record system, health professional, utilization, determinants, Ethiopia, medical record, EMR, EHR, electronic health record, health information technology, systematic review

## Abstract

**Background:**

The strategic plan of the Ethiopian Ministry of Health recommends an electronic medical record (EMR) system to enhance health care delivery and streamline data systems. However, only a few exhaustive systematic reviews and meta-analyses have been conducted on the degree of EMR use in Ethiopia and the factors influencing success. This will emphasize the factors that make EMR effective and increase awareness of its widespread use among future implementers in Ethiopia.

**Objective:**

This study aims to determine the pooled estimate of EMR use and success determinants among health professionals in Ethiopia.

**Methods:**

We developed a protocol and searched PubMed, Web of Sciences, African Journals OnLine, Embase, MEDLINE, and Scopus to identify relevant studies. To assess the quality of each included study, we used the Joanna Briggs Institute quality assessment tool using 9 criteria. The applicable data were extracted using Microsoft Excel 2019, and the data were then analyzed using Stata software (version 11; StataCorp). The presence of total heterogeneity across included studies was calculated using the index of heterogeneity I^2^ statistics. The pooled size of EMR use was estimated using a random effect model with a 95% CI.

**Results:**

After reviewing 11,026 research papers, 5 papers with a combined total of 2439 health workers were included in the evaluation and meta-analysis. The pooled estimate of EMR usage in Ethiopia was 51.85% (95% CI 37.14%-66.55%). The subgroup study found that the northern Ethiopian region had the greatest EMR utilization rate (58.75%) and that higher (54.99%) utilization was also seen in publications published after 2016. Age groups <30 years, access to an EMR manual, EMR-related training, and managerial support were identified factors associated with EMR use among health workers.

**Conclusions:**

The use of EMR systems in Ethiopia is relatively low. Belonging to a young age group, accessing an EMR manual, receiving EMR-related training, and managerial support were identified as factors associated with EMR use among health workers. As a result, to increase the use of EMRs by health care providers, it is essential to provide management support and an EMR training program and make the EMR manual accessible to health professionals.

## Introduction

### Background

Health information technology has transformed and improved health care delivery worldwide. Health information technology has been used for patient administration and management in health care systems. The electronic medical record (EMR) is widely regarded as a critical health information technology tool for improving the quality of medical care [1]. EMRs are computerized patient record systems introduced in the early 1970s to collect, store, and display patient information [2,3]. EMRs can include a variety of clinical services units, such as test ordering, consultation, e-prescription, decision support system, digital imaging, and telemedicine, while protecting patient privacy and confidentiality [4-6].

Implementing the EMR system is the priority agenda in both high-income and resource-limited countries [7]. The adoption of EMRs is a prerequisite for improving clinical decision-making as well as the privacy and security of patients’ information [1]. The perceived benefits that EMRs could provide for the health care system include the following: safety, the organization of patient information, coordination of care, communication, health history, timely access to medical information, and the effectiveness of care [7-9]. Furthermore, evidence shows that EMRs can improve data quality by recording patient information and performing health care functions [8]. This prompted health administrators to develop a program to promote the use of EMRs in the health care system. However, a small proportion of low-income countries have successfully implemented national health information systems.

The Ethiopian Ministry of Health, with the assistance of various nongovernmental organizations, adapted the SmartCare EMR system as a national EMR system for all hospitals and scaled it up to additional hospitals and regions [10,11]. However, individual studies report that this EMR system is underused in Ethiopia, and the system faces sustainability challenges. According to a survey of the comprehensive evaluation of EMR systems in 5 Ethiopian hospitals, only about 31.7% of the participants used EMRs [7]. Similar studies in eastern Ethiopia revealed that EMRs are being used optimally [2]. Another study in the northwestern part of Ethiopia found that only 46.5% of participants used hospital EMR systems [11]. The main reasons for low utilization are implementation challenges and a lack of preimplementation measures, such as EMR readiness, knowledge of EMR, attitude toward EMR, and preimplementation training [2,10,11].

Ethiopia is currently implementing several initiatives to address the abovementioned challenges and strengthen national e-health systems to improve health data availability, accessibility, quality, and use in decision-making processes [12]. The strategic plan calls for an EMR system to streamline data systems and improve the health care delivery [13]. However, only a few comprehensive systematic reviews and meta-analyses are available on the level of EMR use in Ethiopia and the factors that influence its success. As a result, determining the combined level of use and identifying determinants affecting health professionals’ EMR use is critical in confirming its optimal integration and ultimately measuring the benefits within the health care system.

### Objective

This study is unique as it aims to expand our knowledge of the combined level of EMR usage by a health practitioner and offers important recommendations for the effective, efficient, and desirable integration of EMR systems into the Ethiopian health care system.

In our review, we specifically looked into the following questions:

- What is the pooled level of EMR use in Ethiopia?
- What are the determinant factors for EMR use in Ethiopia?

## Methods

### Reporting

This study followed the PRISMA (Preferred Reporting Items for Systematic Reviews and Meta-Analyses) guidelines in its design and reporting (Multimedia Appendix 1) [14].

### Search Strategy and Study Selection

We developed a protocol and searched PubMed, MEDLINE, Web of Sciences, African Journals OnLine, EMBASE, and Scopus to research EMR use and determinants in Ethiopia. To find publications, the following search strategy is used to do extensive searches in web-based databases: [“electronic medical record’ OR ‘electronic health record” OR ‘electronic patient record’ OR ‘Decision Support Systems’] AND [‘determinant’ OR ‘associated factors’ OR ‘barriers’].

### Inclusion and Exclusion Criteria

Studies investigating the utilization and determinants of EMR systems in Ethiopia by the end of June 2022 were considered eligible. Studies that were published in English, in peer-reviewed journals, or as freely accessible full-text publications in the grey literature were all included in this analysis. However, studies without full text and with data that are difficult to extract, studies that are not published in English, studies that do not categorize outcome variables, and studies that do not reflect EMR use in Ethiopia were excluded from this analysis.

### Measurement of the Outcome Variable

The main objectives of this review are to determine the pooled prevalence of EMR use and its determinants. EMR use was assessed based on published literature, with a category of “utilized” or “not utilized.” The review’s second outcome variable sought to uncover factors associated with Ethiopian health workers’ use of EMR systems, which were measured using the odds ratio. The odds ratio for each identified factor was determined using the binary outcome data provided by each primary study.

### Data Extraction and Management

Two authors (MDT and SMW) used Microsoft Excel to extract all the essential parameters independently. The first author’s last name, year of publication, region, study area, study design, study population, sample size, percentage of EMR use with standard error, and determinant factors that affect utilization with the standard error were all extracted from each study. The disagreements between the two authors were resolved through discussion.

### Quality Appraisal of the Individual Studies

Two authors appraised each study’s quality independently (MDT and TMY). To assess the quality of each included study, we used the Joanna Briggs Institute quality assessment tool using 9 criteria [15]. The tool mainly included (1) an appropriate sample frame; (2) an appropriate sampling strategy; (3) an adequate sample size; (4) a description of the study subjects and setting; (5) data analysis conducted with sufficient coverage; (6) valid methods for condition identification; (7) the condition measured in a standard, reliable way for all participants; (8) appropriate statistical analysis; and (9) an adequate response rate. Each item was given a rating of “yes,” “not reported,” or “not appropriate.” Finally, the total quality score was assigned based on the number of “yes” responses per study. Papers with a rating of 5 or above out of 9 were included in the final review (Multimedia Appendix 2).

### Data Processing and Analysis

The relevant data were extracted using Microsoft Excel 2019. The data were then analyzed using Stata software (version 11; StataCorp). The pooled size of EMR use was estimated using a random effect model with a 95% CI [16]. The percentage of total variation across studies was calculated using the index of heterogeneity *I*^2^ statistics [17]. Due to the heterogeneity of the included studies (*I*^2^>75% and *P*<.05), the data were divided into subgroups according to the study region and year of publication. This was due to the highly diverse study regions and publication years of the included research. As a result, the random differences between the point estimations in the primary research are reduced. Researchers employed Egger’s regression test and funnel plot analysis to identify publication bias [18,19]. *P*<.05 was considered a statistically significant publication bias in Egger’s test.

## Results

### Search Results

**Figure 1:**
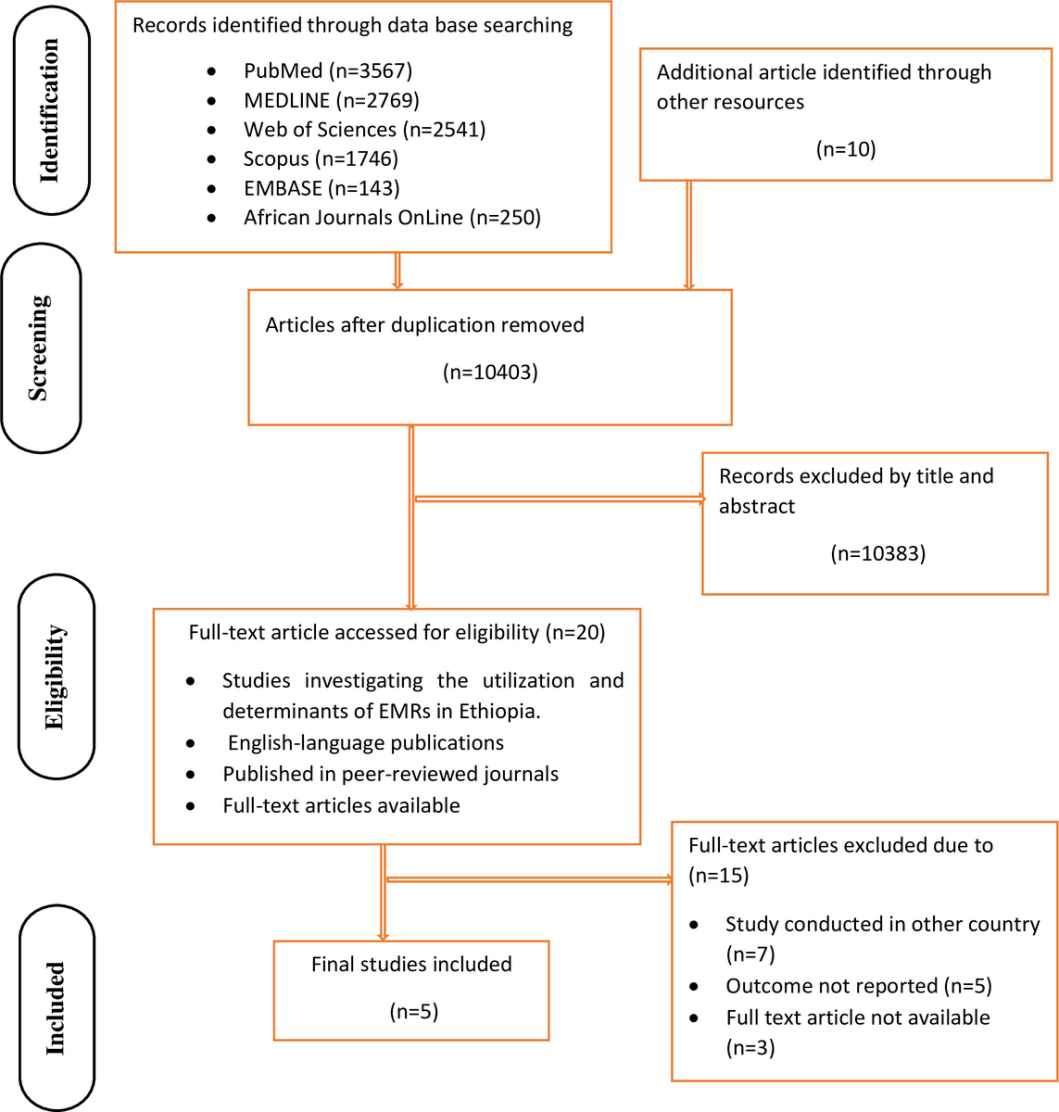
PRISMA (Preferred Reporting Items for Systematic Reviews and Meta-Analyses) flow chart displaying the selection process of included studies.

A total of 11,026 articles on the use and determinants of EMRs in Ethiopia were found in PubMed, MEDLINE, Web of Sciences, African Journals OnLine, EMBASE, and Scopus. From the total number of retrieved studies, 623 papers were removed due to duplication, and 10,383 publications were excluded after being evaluated based on their titles and abstracts. The remaining 20 full-text publications were assessed for eligibility, with 15 articles further excluded based on the inclusion and exclusion criteria. Finally, only 5 publications were included in the final meta-analysis based on the predefined criteria and quality assessment (Figure 1).

### Characteristics of Included Studies

This meta-analysis and systematic review included a total of 2439 health professionals. The number of studies with the smallest and largest sample sizes was 412 and 606, respectively. Among the included primary studies, 2 studies were undertaken in the eastern Ethiopia [2,20], 1 in northwest Ethiopia’s Amhara region [11], 1 in the Tigray region [21], and 1 in Ethiopia’s capital, Addis Ababa [7]. As shown in Table 1, these 5 original studies were published between early December 2014 and November 2021. All included studies used an institutional-based cross-sectional study design to estimate the use of EMR systems, as shown in Table 1.

**Table 1:** Summary of primary cross-sectional studies included in the meta-analysis of the use of electronic medical records among health professionals in Ethiopia, 2022.

Author and publication year	Region	Study area	Sample size	Magnitude	Quality^a^
Oumer et al [2], 2021	Harari region and Dire Dawa	Eastern Ethiopia	412	67.7	9
Mekonnen et al [20], 2021	Harari region	Harari Regional State	498	42.3	7
Biruk et al [11], 2014	Amhara	Northwest Ethiopia	606	46.5	9
Yehualashet et al [21], 2015	Tigray	Ayder Referral Hospital	501	71	8
Tilahun et al [7], 2015	Addis Ababa	Addis Ababa	422	31.7	9

^a^To assess the quality of each included study, we used the Joanna Briggs Institute quality assessment tool using 9 criteria.

### The Pooled Utilization of EMR System in Ethiopia

**Figure 2:**
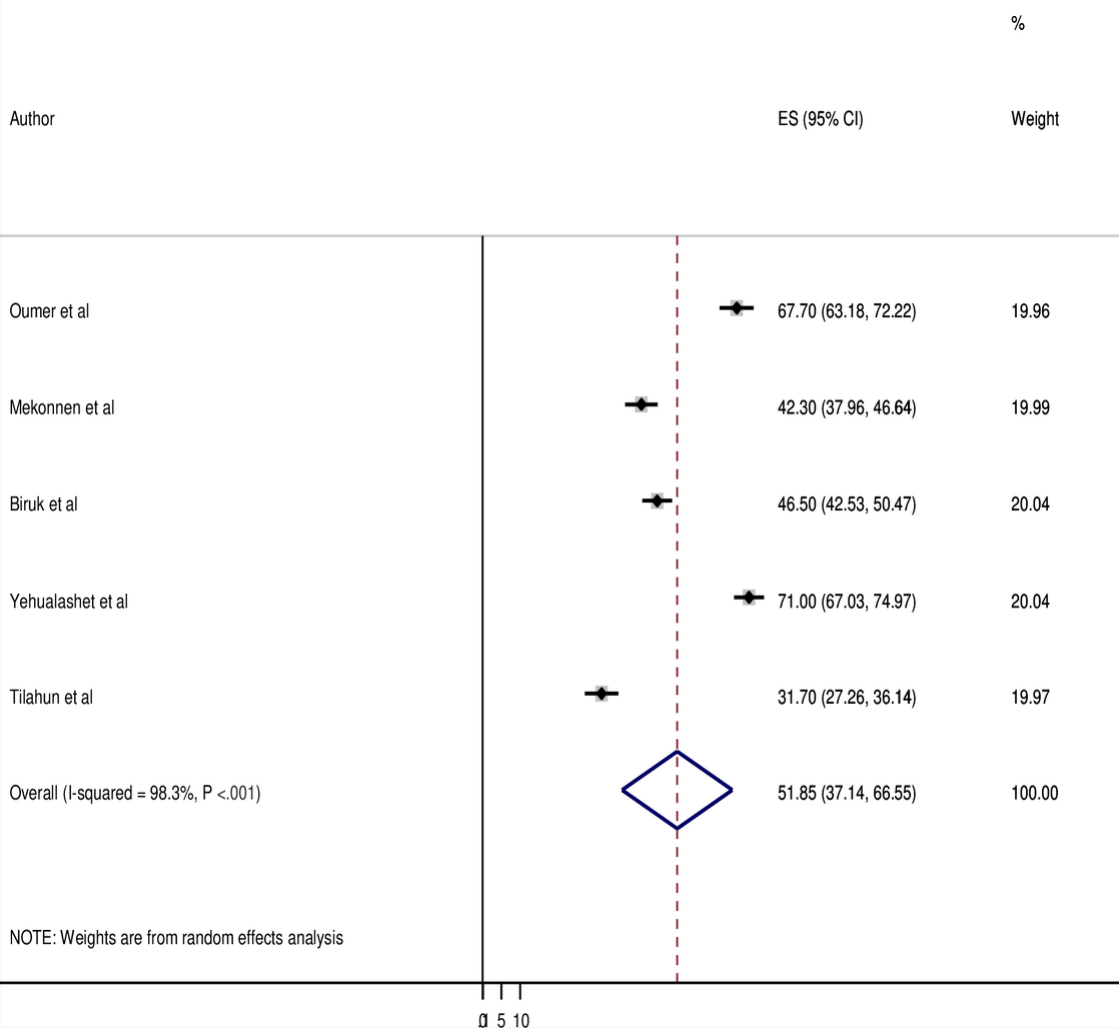
Forest plot displaying a pooled estimate of electronic medical record use among health professionals in Ethiopia. ES: Effect Size.

The pooled estimate of EMR use in Ethiopia from 5 studies [2,7,11,20,21] was 51.85% (95% CI 37.14%-66.55%; Figure 2). The included studies were found to be heterogeneous (*I*^2^>75% and *P*<.05) [22]. Subgroup analysis is done based on the study location and publication year due to high heterogeneity across the included studies (*I*^2^=98.3% and *P*<.001; Figure 2). According to the subgroup study, the northern Ethiopia region ranked highest in EMR use (58.75%), followed by the Eastern portions of Ethiopia (54.99%) and the Addis Ababa region (31.70%; Table 2). Furthermore, disparities in publication time were identified, with current publications on the use of EMRs being higher (54.99%) than the research published before 2016 (49.75%), as shown in Table 2.

**Table 2:** Subgroup analysis by study location and publication year of electronic medical record use among health professionals in Ethiopia.

Variable and subgroup			Number of studies		Sample size, n		Prevalence (95% CI)		*I*^2^ (%)		*P* value
**Study location**											
	Eastern Ethiopia	2		910		54.99 (30.10-79.88)		98.4		<.001	
	Northern Ethiopia	2		1107		58.75 (34.74-82.76)		98.6		<.001	
	Addis Ababa	1		301		31.70 (27.26-66.55)		—^a^		—	
**Year of publication**											
	Before 2016	3		1529		49.75 (27.50-72.00)		98.9		<.001	
	After 2016	2		910		54.99 (30.10-79.88)		98.4		<.001	

^a^Not applicable.

### Sensitivity Analysis and Publication Bias

**Figure 3:**
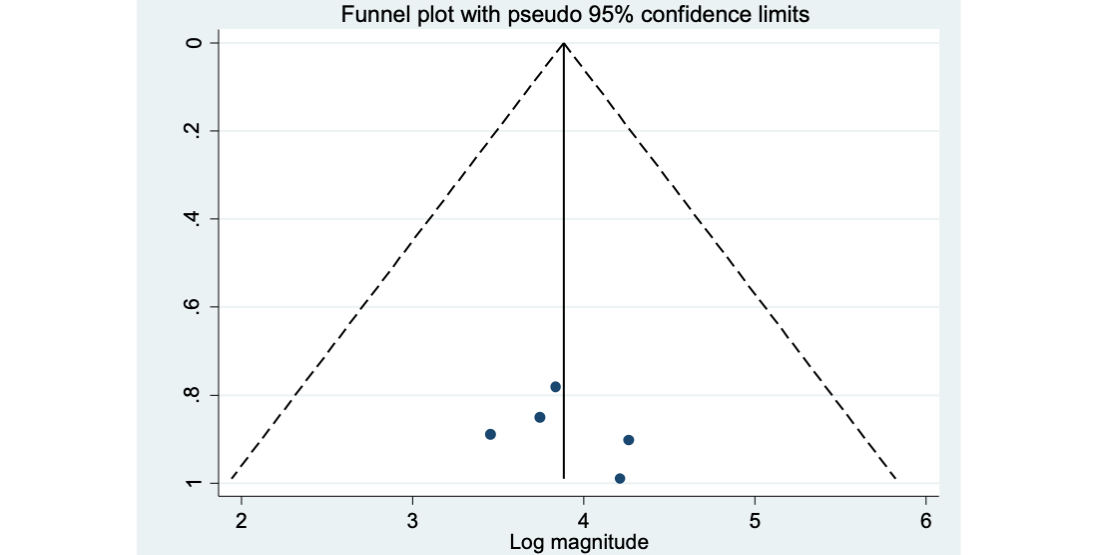
Funnel plot to test publication bias of the 5 included studies.

Sensitivity analysis revealed that the overall effect sizes remained stable with the deletion of any of the studies from the analysis without a notable improvement in heterogeneity (Table 3). A funnel plot and Egger’s regression test were used to investigate potential publication bias. As a result, the funnel plot is symmetric, indicating no publishing bias because all of the research falls inside the triangular region (Figure 3). Furthermore, Egger’s regression test results revealed no evidence of publication bias (*P*=.30; Table 4).

**Table 3:** Sensitivity analysis results for the 5 studies.

Study omitted	Estimates (95% CI)	Hetrogenity	
		*I*^2^ (%)	*P* value
Oumer et al [2], 2021	47.89 (31.43-64.35)	98.4	<.001
Mekonnen et al [20], 2021	54.23 (36.36-72.10)	98.6	<.001
Biruk et al [11], 2014	53.18 (34.30-72.07)	98.7	<.001
Yehualashet et al [21], 2015	47.04 (32.82-61.27)	97.7	<.001
Tilahun et al [7], 2015	56.87 (42.56-71.19)	97.9	<.001
Combined	51.85 (37.14-66.55)	98.3	<.001

**Table 4:** Egger’s test for publication bias of the 5 studies.

SE of the effect size	Coefficient	SE	*t* value	*P* value	95% CI
Slope	0.115799	0.4459117	0.26	.80	–0.9386145 to 1.170213
Bias	2.147455	1.909221	1.12	.30	–2.367135 to 6.662046

### Factor Associated With the Use of EMR Systems

Some of the factors associated with the use of EMRs were quantitatively pooled in this systematic review and meta-analysis. In contrast, others were not because the independent variables were not consistently categorized about the use of EMRs.

**Figure 4:**
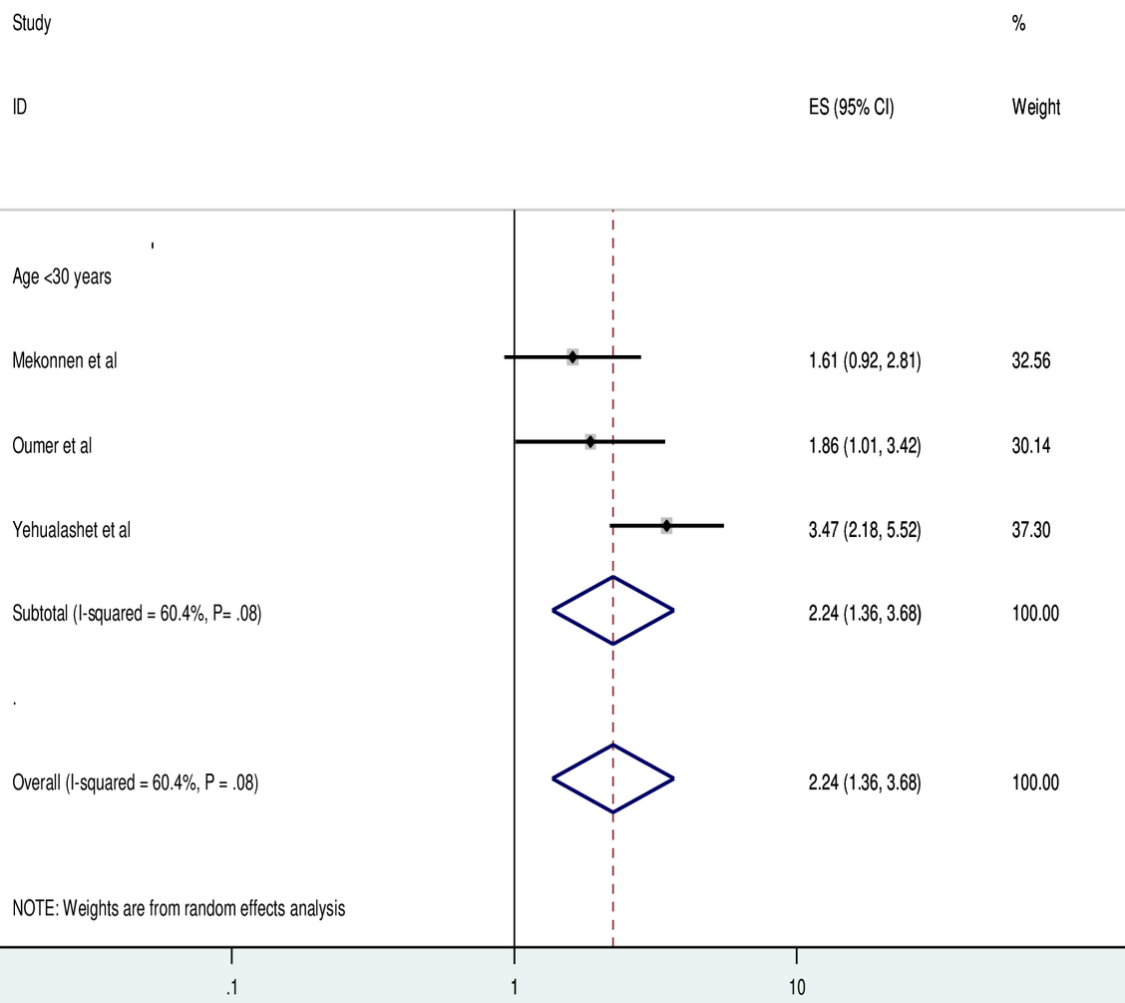
Forest plot displaying the association between younger age group and use of electronic medical records among health professionals in Ethiopia. ES: Effect Size.

Three studies indicated that health professionals who were younger (age groups <30 years) were 2.24 times (adjusted odds ratio [AOR]=2.24, 95% CI 1.36-3.68) more likely to use EMR compared to those whose age group was greater than or equal to 30 years. The included studies were characterized by the presence of heterogeneity (*I*^2^=60.4%; *P*=.08). Hence a random effect model analysis was performed in this meta-analysis (Figure 4).

**Figure 5:**
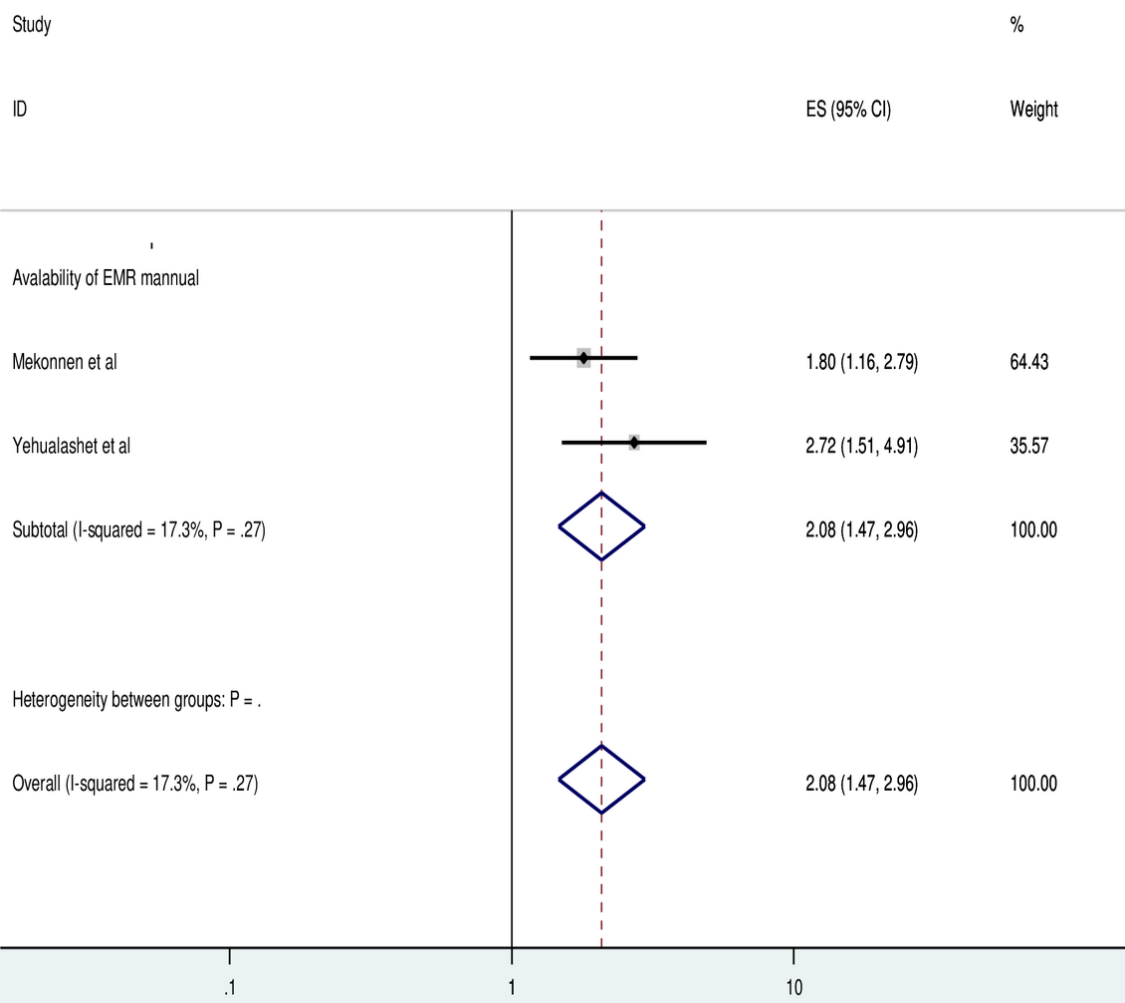
Forest plot displaying the association between availability of electronic medical record (EMR) manual and the use of EMR among health professionals in Ethiopia. ES: Effect Size.

Two studies showed that the presence of an EMR manual has a significant association with the use of EMR systems. The odds of EMR use were 2.86 times (AOR=2.08, 95% CI 1.47-2.96) higher for health care professionals with EMR manuals compared to those without them. The included studies in this meta-analysis did not exhibit any heterogeneity (*I*^2^=17.3%; *P*=.27). Consequently, a fixed-effect model analysis was performed (Figure 5).

**Figure 6:**
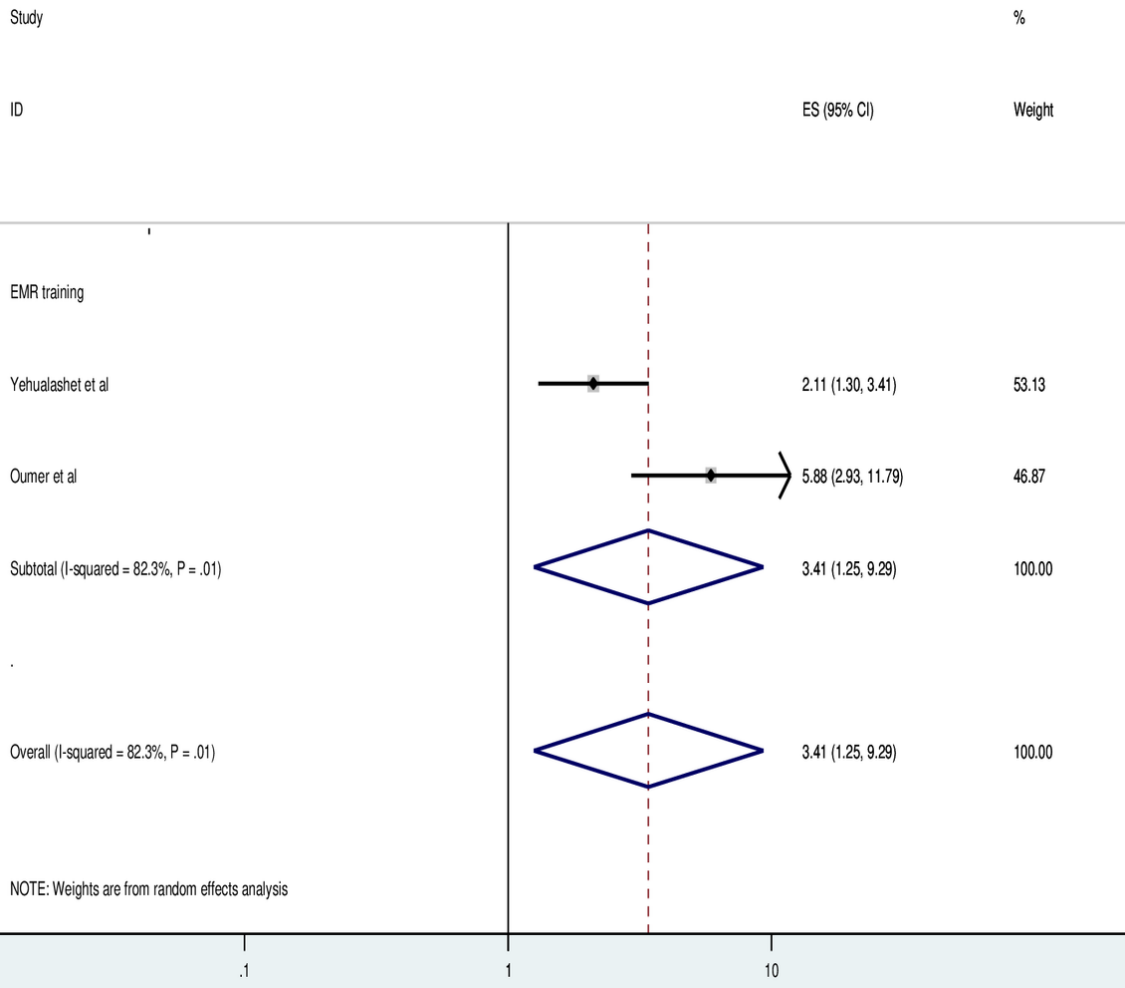
Forest plot displaying the association between electronic medical record (EMR) training and the use of EMR among health professionals in Ethiopia. ES: Effect Size.

Two studies showed that training related to EMRs has a significant association with the use of EMR systems. The odds of using EMRs were 3.41 times (AOR=3.41, 95%CI 1.25-9.29) higher for health professionals who routinely received EMR training compared to those who did not. Random effects model analysis was carried out in this meta-analysis because the included studies were characterized by the existence of heterogeneity (*I*^2^=82.3%; *P*=.02; Figure 6).

**Figure 7:**
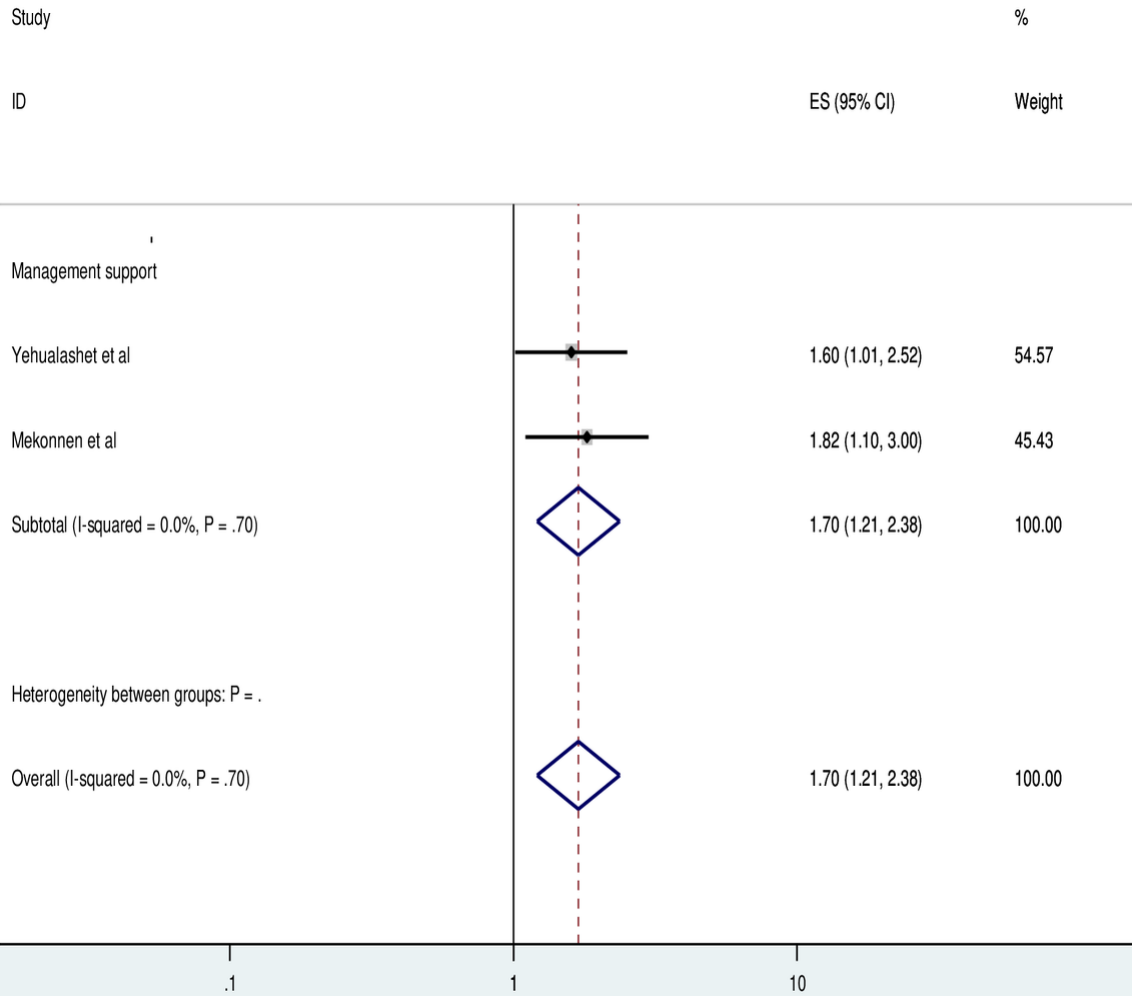
Forest plot displaying the association between availability of management support and the use of electronic medical records among health professionals in Ethiopia. ES: Effect Size.

Furthermore, 2 studies indicated a significant association between using an EMR system and receiving managerial support. Health care professionals who got managerial support were 2.86 times (AOR=1.70, 95% CI 1.21-2.38) more likely to use EMR systems compared to those who did not get managerial support. There was no heterogeneity among the papers included in this meta-analysis (*I*^2^=0%; *P*=71). As a result, a fixed-effect model analysis was carried out (Figure 7).

## Discussion

### Principal Findings

This systematic review and meta-analysis investigated the use and determinants of the EMR system among health professionals in Ethiopia. Results revealed that the pooled estimate of EMR system use among health care professionals in Ethiopia was 51.85% (95% CI 37.14%-66.55%). We carried out a subgroup analysis based on the study site, where the studies were conducted. In the subgroup study, the northern Ethiopia region had the greatest rate of EMR utilization (58.75%), followed by the eastern parts of Ethiopia (54.99%). Similarly, we also carried out a subgroup analysis based on the year of publication of the original studies. We discovered disparities in publication timing, with current publications on the use of EMRs being higher in percentage (54.99%) than the studies published before 2016 (49.75%).

Furthermore, this analysis was conducted to identify determinants of EMR utilization in Ethiopia. The results showed that health care professionals younger than 30 years, health care professionals with access to an EMR manual, health care professionals with EMR-related training, and health care professionals with managerial support were found to have a positive association with the use of EMR in Ethiopia.

### Comparison With Prior Work

Despite the lack of a meta-analysis on this topic of research, the use of the EMR system presented in this study is consistent with earlier individual studies conducted in Saudi Arabia (52%) [23]. Our results show a slightly lower rate of EMR use compared to those of studies done in Malawi on central hospitals, which showed that 68.8% of health workers used EMRs for collecting and analyzing clinical data [24]. However, the results of this study show a considerably lower rate of EMR use compared to those of studies conducted in industrialized nations, where the use of EMRs was 98% in Sweden, 88% in France, 88% in Germany, and 70% in Switzerland [25]. The discrepancy may be caused by disparities in information and communications technology infrastructure between lower- and higher-income nations, where in the case of lower-income countries, there is a power outage, limited access to standby generators, inadequate maintenance, and technical issues [21]. Furthermore, the lack of a standardized EMR system in Ethiopia and the health professionals’ inadequate understanding and attitude toward EMRs may be contributing factors to Ethiopia’s lower EMR use [26].

The northern Ethiopia region had a greater rate of EMR utilization, followed by the eastern part of Ethiopia, according to the subgroup analysis based on the study sites. This disparity could result from Ethiopia’s northern region serving as a test site for implementing EMR systems. EMRs are used more frequently in the Ayder specialized hospital in northern Ethiopia than in the other studies that were taken into account [21]. Furthermore, the University of Gondar specialized hospital in northern Ethiopia served as a center of expertise for implementing EMRs [12].

The subgroup analysis based on publication year also revealed that studies published before 2016 showed lower EMR usage than recent studies. This could be attributed to the “Information Revolution,” one of the transformative goals of the current Ethiopian federal ministry of health [27]. As a result, most hospitals have implemented EMRs and have mentorship and capacity-building programs for health care professionals regarding the use of health information and data quality [12,27].

This review showed that younger health professionals were more likely to use EMR than people in older age groups. Previous investigations have also supported these findings [2,21]. This might be because younger medical professionals are more open to adopting new technologies and have a better comprehension of information and communications technology than their older counterparts [28-30]. This implied that special attention should be paid to older health care professionals to boost the acceptance of the new health information technology.

This study also showed a strong link between using EMR systems and accessing an EMR manual. This suggests that health providers needed access to the EMR guidelines to promote the usage of EMR systems. Substantial evidence from various places supports this justification [28-30]. Our analysis also revealed that receiving management assistance has a significant association with the use of EMR systems. Previous research has shown that managerial support is the foundation for increasing the use of EMRs by health care workers [11,31]. This suggests that health administrators must work very hard to enhance the usage of EMRs and encourage their staff to use EMRs to make data-driven decisions that will raise the quality of health services.

Furthermore, our findings showed a strong correlation between receiving EMR training and using EMR systems. This result is consistent with earlier studies that discovered EMR system training positively impacted using the EMR system [32-34]. According to this finding, the health care system’s adoption of health information technology may be strongly impacted by ongoing EMR and basic computer training. This recommends that the Ethiopian Ministry of Health should get ready to give thorough end-user training packages for medical staff to increase the level of EMR use and ensure its successful implementation.

### Limitations

We are aware that there are certain limitations to this review. The review’s primary challenge is the small number of included studies. Additionally, because of the varied categorization of factors in the included study, the pooled odds ratio for all variables associated with using EMRs by health workers was not evaluated. Furthermore, since all of the included papers were facility-based cross-sectional studies, the quality of the evidence and the generalization of the findings may be diminished. However, we tried to produce high-quality evidence by evaluating each included study’s quality using 9 criteria from the Joanna Briggs Institute’s quality evaluation tool.

### Conclusions

The use of EMR systems in Ethiopia is relatively low. This study provides strong evidence for future implementers to pay close attention to improving health professionals’ use of EMRs after implementation. This can be accomplished by making the EMR manual available to health practitioners, offering an EMR training program, and providing managerial support.

## Appendix

Multimedia Appendix 1 PRISMA (Preferred Reporting Items for Systematic Reviews and Meta-Analyses) checklist.

Multimedia Appendix 2 Quality score.

## Abbreviations

AOR: adjusted odds ratio
EMR: electronic medical record
PRISMA: Preferred Reporting Items for Systematic Reviews and Meta-Analyses

## Data Availability

The data analyzed during this meta-analysis and supplementary information are available in the published document.
