# Supplementary material for: Electronic Medical Record System Use and Determinants in Ethiopia: Systematic Review and Meta-Analysis
Source: Interact J Med Res. 2023 Jan 11;12:e40721. doi: 10.2196/40721 (PMC9878362; doi:10.2196/40721)
Supplement: Multimedia Appendix 2 [file ijmr_v12i1e40721_app2.docx]

Table_S2 Quality assessment of utilization and determinants of EMRs among health professionals in Ethiopia included studies in this systematic review and meta-analysis.

| **Author, year of**  **Study** | **Q1** | **Q2** | **Q3** | **Q4** | **Q5** | **Q6** | **Q7** | **Q8** | **Q9** | **Total score (9%)** |
| --- | --- | --- | --- | --- | --- | --- | --- | --- | --- | --- |
| Oumer et.al, 2021 | Y | Y | Y | Y | Y | Y | Y | Y | Y | 9 |
| Biruk et.al, 2014 | Y | Y | Y | Y | Y | Y | Y | Y | Y | 9 |
| Mekonnen et.al, 2021 | NR | Y | NA | Y | Y | Y | Y | Y | Y | 7 |
| Tilahun et.al, 2015 | Y | Y | Y | Y | Y | Y | Y | Y | Y | 9 |
| Yehualashet et.al, 2015 | Y | Y | Y | Y | Y | Y | NR | Y | Y | 8 |

**Key:** **Y**= Yes; **NR**= Not reported, **NA**=Not appropriate

**Question codes:**

1. Was the sample frame appropriate to address the target population?

2. Were study participants sampled in an appropriate way?

3. Was the sample size adequate?

4. Were the study subjects and the setting described in detail?

5. Was the data analysis conducted with sufficient coverage of the identified sample?

6. Were valid methods used for the identification of the condition?

7. Was the condition measured in a standard, reliable way for all participants?

8. Was there appropriate statistical analysis?

9. was the response rate adequate, and if not, was the low response rate managed appropriately?
